# HtrA1 expression and the prognosis of high-grade serous ovarian carcinoma: a cohort study using digital analysis

**DOI:** 10.1186/s13000-018-0736-6

**Published:** 2018-08-21

**Authors:** Andréanne Gagné, Bernard Têtu, Michèle Orain, Stéphane Turcotte, Marie Plante, Jean Grégoire, Marie-Claude Renaud, Isabelle Bairati, Dominique Trueel

**Affiliations:** 1 Laval University Cancer Research Center, Hôtel-Dieu-de-Québec, Centre Hospitalier Universitaire (CHU) de Québec, 11 Côte du Palais, Québec, Québec G1R 2J6 Canada; 2 Anatomic Pathology and Cytology Department, Hôpital du St-Sacrement, Centre Hospitalier Universitaire (CHU) de Québec, Laval University, 1050 Chemin Ste-Foy, Québec, Québec G1S 4L8 Canada; 3 Gynecologic Oncology Division, Centre Hospitalier Universitaire (CHU) de Québec, L’Hôtel-Dieu-de-Québec, 11 Côte du Palais, Québec, Québec G1R 2J6 Canada; 4 Department of Pathology, Hôpital Saint-Luc, Centre Hospitalier Universitaire de Montréal, 058, rue Saint-Denis, Montréal, Québec H2X 3J4 Canada; 5 The Research Centre of the University of Montreal Teaching Hospital (CR-CHUM)/Montreal Cancer Institute, 900 Rue St-Denis, Montreal, Quebec H2X 0A9 Canada; 6 Department of Pathology and Cellular Biology, University of Montreal, 2900, boulevard Édouard-Montpetit, Montreal, Quebec H3T 1J4 Canada; 7 Department of Pathology, Hôpital du St-Sacrement, Centre Hospitalier Universitaire de Québec, 1050, Chemin Ste-Foy, Québec, Québec G1S 4L8 Canada

**Keywords:** Ovarian high grade serous carcinoma, High temperature requirement factor A1, Immunohistochemistry, Digital image analysis, Prognosis

## Abstract

**Background:**

The expression of high temperature requirement factor A1 (Htra1) has been reported to be decreased in ovarian carcinoma, but its prognostic effect remains undetermined.

**Methods:**

We evaluated the impact of HtrA1 downregulation in tumoral tissues on cancer progression and death in women with serous ovarian carcinoma. HtrA1 staining was performed on tissue microarrays (TMA) comprised of tumor samples from a cohort of 106 women who were diagnosed with primary high-grade serous ovarian carcinoma and receiving standard treatment at the Québec University Hospital between 1993 and 2006. HtrA1 expression was assessed visually (percentage of positive nuclei) and by digital image analysis (percentage of positive area). Cox regression multivariate models included standard prognostic factors and were used to estimate adjusted hazard ratios (aHR) for progression or death in the cohort.

**Results:**

By visual analysis, a low percentage of HtrA1-positive nuclei (< 10% vs ≥10%) tend to be associated with a lower risk of progression (aHR = 0.71; 95% Confidence interval (CI) = 0.46–1.09; *P* = 0.11) and mortality (aHR = 0.65; 95% CI = 0.41–1.04; *P* = 0.07). Low nuclear HtrA1 expression assessed by digital image analysis (< median % vs ≥ median %) showed a significant association with lower risk of progression (aHR = 0.62; 95% CI = 0.40–0.95; *p* = 0.03) and death (aHR = 0.60; 95% CI = 0.38–0.95; *p* = 0.03).

**Conclusion:**

Altogether, our results demonstrate that nuclear downregulation of HtrA1 is associated with a better prognosis in women with high grade serous ovarian carcinoma.

## Background

Worldwide, ovarian carcinoma is the second most frequent neoplasm of the female genital system [1, 2]. In the United States, ovarian carcinoma was the most lethal neoplasm of the female genital system in 2017 [3]. The high mortality rate of ovarian cancer can be explained by the fact that about 80% of women are diagnosed with advanced stage disease (FIGO stage III and IV) [4]. Early symptoms of the disease are non-specific, leading to a delay in the diagnosis [4]. Most epithelial ovarian cancers are identified as high-grade serous carcinomas, which have been associated with specific genetic profiles [4].

We have previously shown that many matrix metalloproteinases (MMPs) are associated with the prognosis of ovarian carcinoma [5–7]. Apart from MMPs, other proteases have also been proposed for prognostic potential in ovarian carcinoma. The protease high temperature requirement factor A1 (HtrA1) is expressed in the vast majority of healthy tissues in the human body, including gynecologic tissues and the ovary [8]. HtrA1 was previously demonstrated to be associated to anoikis, a form of apoptosis [9, 10] and to cellular migration [11]. In human ovarian cancer tissues, the expression levels of HtrA1 mRNA and protein are significantly lower than in normal epithelial tissues [10, 12, 13]. Although the prognostic effect of HtrA1 in ovarian carcinoma is unknown, HtrA1 downregulation has been reported in association with resistance to cisplatin [14, 15]. The downregulation of HtrA1 has also been associated with poor prognosis in stomach, breast and liver cancers [14, 16–18]. Altogether, these data reveal that HtrA1 has an important effect in cancer that could potentially be targeted for therapy. Accordingly, the purpose of this study is to evaluate the impact of HtrA1 downregulation in tumoral tissues on cancer progression and death in women with high-grade serous ovarian cancer.

## Methods

### Cohort

Eligible women had a diagnosis of primary high-grade serous ovarian cancer and received standard treatment that involved debulking surgery and adjuvant chemotherapy when indicated [19, 20]. Women were recruited between 1993 and 2006 at l’Hôtel-Dieu de Québec of the Centre Hospitalier Universitaire de Québec (CHU de Québec), Canada. All the women in the cohort gave written consent to donate cancer tissues for the tumor bank. The local ethics committee of CHU de Québec approved the study (approval number: 2012-404, H09-06-052).

### Clinical data

Clinical characteristics (age, pre-operative serum CA-125 levels, FIGO stage and presence of residual tumor) were collected from medical files. Two pathologists (BT, DT) confirmed tumor histologic type and grade after slide review. The surgical and chemotherapy modalities were also recorded.

Cancer progression was evaluated by the CA-125 level and the Response Evaluation Criteria in Solid Tumors (RECIST), as suggested in the Gynaecologic Cancer InterGroup (GCIG) criteria [21, 22]. When progression was documented by both radiology (RECIST) or CA-125 levels, the recorded date of progression was the earliest date among the two events. Documents from the *Ministère de la Santé et des Services Sociaux du Québec* were accessed to obtain data on patients death. The cohort was followed until August 2012.

### Tissue microarrays (TMA) and immunohistochemistry

Formalin-fixed and paraffin-embedded (FFPE) tumor samples from the tumor bank were reviewed by a pathologist (DT) to select representative sections and highlight specific zones for puncture. Three 0.6 mm tumor cores were taken from each tumor and embedded into a paraffin block using a tissue arrayer (Beecher Instruments Tissue Microarray Technology, Estigen, Sun Prairie, WI, USA). Tumor cores were randomized in the TMA.

Antibody specificity for HtrA1 was verified by performing Western blots with protein extracts from 6 breast and ovarian cell lines (MCF7, TOV2223G, TOV1946, TOV112D, OV90, TOV81D) [23, 24]. Thirty micrograms of proteins from each cell line were separated by SDS-polyacrylamide gel electrophoresis, and then transferred on nitrocellulose membranes. Membranes were blocked with 5% milk in phosphate-buffered saline (PBS)/0.1% Tween for 60 min at room temperature (RT). Incubation followed with the rabbit polyclonal anti-HtrA1 primary antibody (cat no. ab38611, ABCAM), diluted 1/200 in 2.5% milk in PBS/0.01% Tween at 4 °C overnight. Following the manufacturer’s instruction, detection was performed with enhanced chemiluminescence (cat no. RPN3243, GE Healthcare). Monoclonal antibody against beta-actin (cat no. ab6276, ABCAM) was used as a control.

Immunohistochemistry was performed on 4 μm thick sections from each TMA block and two sections from an ovarian tumor with known high HtrA1 levels as a positive control for HtrA1. Tissue sections were deparaffinized with toluene and rehydrated in graded ethanol, then pretreated in 10 mM citrate buffer, pH 6. Tissue sections were incubated for 5 min in 3% hydrogen peroxide. Biotin Blocking System (cat no. X0590, DAKO) was then used to limit non-specific binding due to the presence of biotin in ovarian carcinoma tissues. Sections were incubated for 20 min in blocking serum at RT, and then treated with the rabbit polyclonal anti-HtrA1 antibody at a 1/75 dilution in 1% bovine serum albumin (BSA) in PBS for 1 h at RT. Detection was achieved by the avidin-biotin method using the Super Stain HRP kit (cat no. IDST1007, IDLabs, London, ON, Canada), followed by chromogenic staining (3, 3′- diaminobenzidine; cat no. BP1111, IDLabs) and counterstain (Harris haematoxylin). As negative control, we used normal smooth muscle which is recognized to stain negatively for HtrA1. We also used the positive control which was incubated in 1% BSA in PBS instead of the primary antibody.

### Visual evaluation

All TMA slides were digitized with a slide scanner (NanoZoomer 2.0-HT, Hamamatsu, Bridgewater, NJ, USA) and were visualised with an image viewer software (NDP view, Hamamatsu). HtrA1 expression was assessed visually by evaluating the percentage of positive nuclei in the samples on an interval scale with 10 categories from 0 to 100% (0–9%, 10–19%, 20–29% etc). The same increments were used to evaluate the percentage of cells with cytoplasmic staining. Cores were evaluated only if more than 30% of the surface was composed of tumor. A patient’s sample was excluded if less than 2 of 3 cores could not be evaluated. Slides were assessed by a trained medical student (AG) and revised by a pathologist (DT), both blinded to clinical information. Disagreements were resolved by a senior pathologist (BT) who was blinded to the results of the first slide assessment.

### Digital evaluation

CaloPix image management program (TRIBVN, Châtillon, France), was used for digital analysis. The Ilastik 5.0 Interactive Learning and Segmentation Toolkit is integrated into CaloPix and permits tissue recognition. The program was manually trained to isolate serous ovarian tumor tissues from their environment (stroma and other structures) and to identify tumor cells, generating “immunosurface” and “immunoobject” algorithms to calculate the percentages of positive cytoplasms and nuclei respectively. Digital analysis was performed after image compression equivalent to 10× resolution. After digital processing, all samples on each TMA were revised visually (AG). Infrequently, if tissue recognition provided by Ilastik algorithm was unsatisfactory, a manual segmentation was used.

### Statistical analysis

Descriptive analyses were undertaken to describe the distribution of HtrA1 staining according to patients’ characteristics. For each patient, the average of the percentage of HtrA1-positive cells was calculated for both cytoplasm and nuclei. Chi-square tests and Fisher exact tests were conducted to evaluate the associations between the expression of HtrA1 and standard prognostic factors. Accuracy between visual and digital analysis was measured. Kaplan-Meier curves and log-rank tests were performed to estimate the association between HtrA1 and progression or death of women with high grade serous ovarian carcinoma. Time to progression or death was calculated from the date of debulking surgery to the date of the event. If no event occurred, the date of the last CA-125 measurement or medical visit was recorded. Cox proportional hazards models were built to estimate crude and adjusted hazard ratios (aHR) and their 95% confidence interval (CI). HR estimates were adjusted for FIGO stage (III-IV versus I-II), age at diagnosis (continuous) and pre-operative serum CA-125 levels (dichotomized according to the median value, ≥ 681.5 pmol/L versus < 681.5 pmol/L). Residual tumor was not included in the model as this variable was strongly correlated to the FIGO stage (*p* < 0.0001). All statistical analyses were performed using SAS 9.2 software (SAS Institute, Cary, NC, USA) and all tests were two-sided.

## Results

### Cohort

**Fig. 1:**
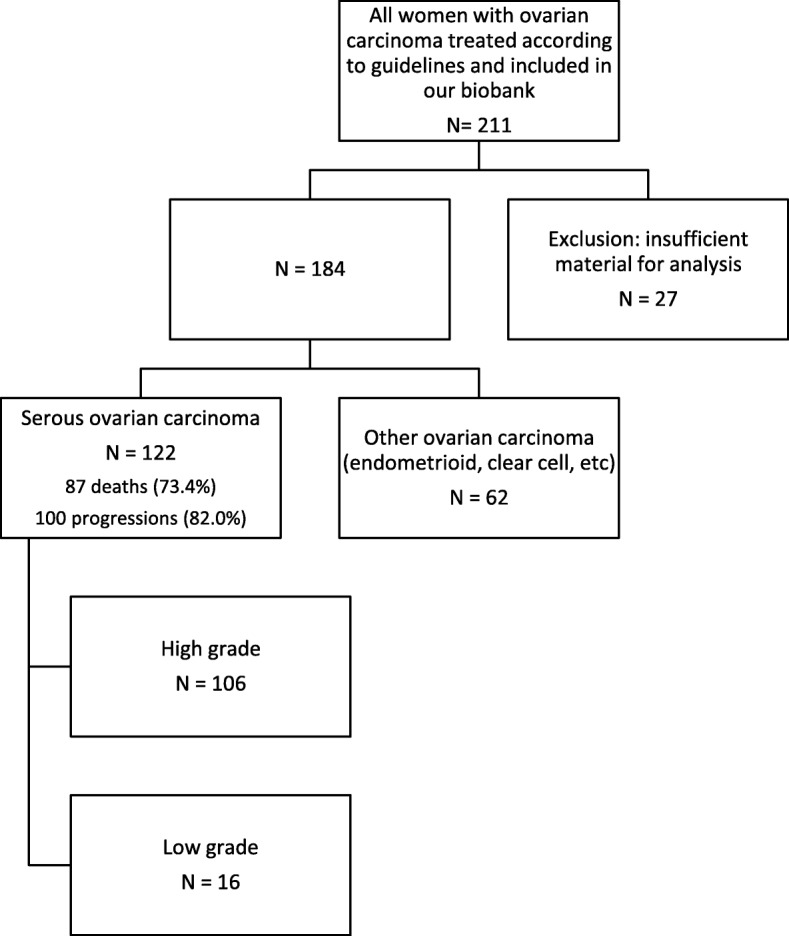
Study flow chart

Our final cohort included 106 women with high grade serous ovarian carcinoma and with available histopathological material (Fig. 1). Most women had advanced stage disease (FIGO stage III-IV; *n* = 98, 92.4%) with residual tumor after surgery (*n* = 88, 83.0%) (Table 1). At 5 years, progression-free survival was 19.05% (95% CI: 12.21–27.06) and survival was 44.26% (95% CI: 34.65–53.42). Medians of progression free survival and death were 1.38 years (95% CI, 1.19–1.57) and 4.16 years (95% CI: 3.31–5.47), respectively.

**Table 1:** Clinicopathological characteristics of the patients with high grade serous ovarian cancer

Characteristics	Patients (*n* = 106)
Average age at diagnosis (standard deviation)	61.4 (10.7)
Median pre-operative serum CA-125 (pmol/L) – [interquartile range]	681.5 [328.0; 1996.0]
FIGO Stage – n (%)	
I-II	8 (7.6)
III-IV	98 (92.4)
Residual tumor - n (%)	
None	18 (17.0)
> 0 cm	88 (83.0)
Adjuvant chemotherapy	
Yes	106 (100.0)
Carboplatin + others	97 (91.5)
Carboplatin + taxol	90 (84.9)

### Immunohistochemistry

**Fig. 2:**
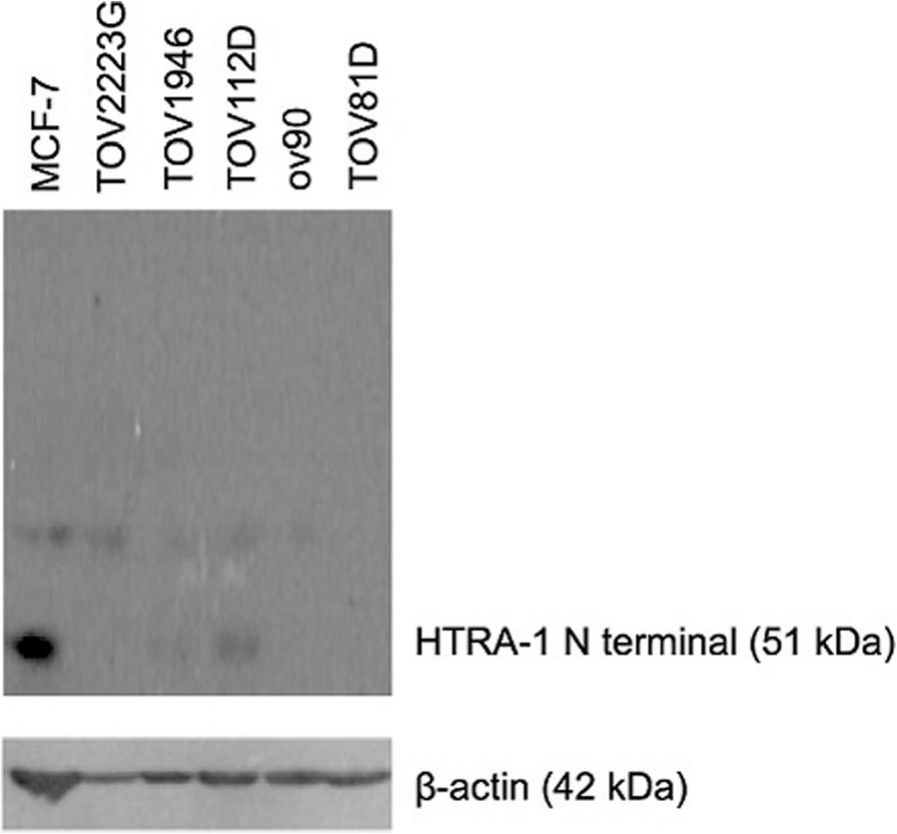
Specificity of anti-HtrA1 antibody for HtrA1 expression demonstrated by Western blot analysis of 6 cell lines

**Fig. 3:**
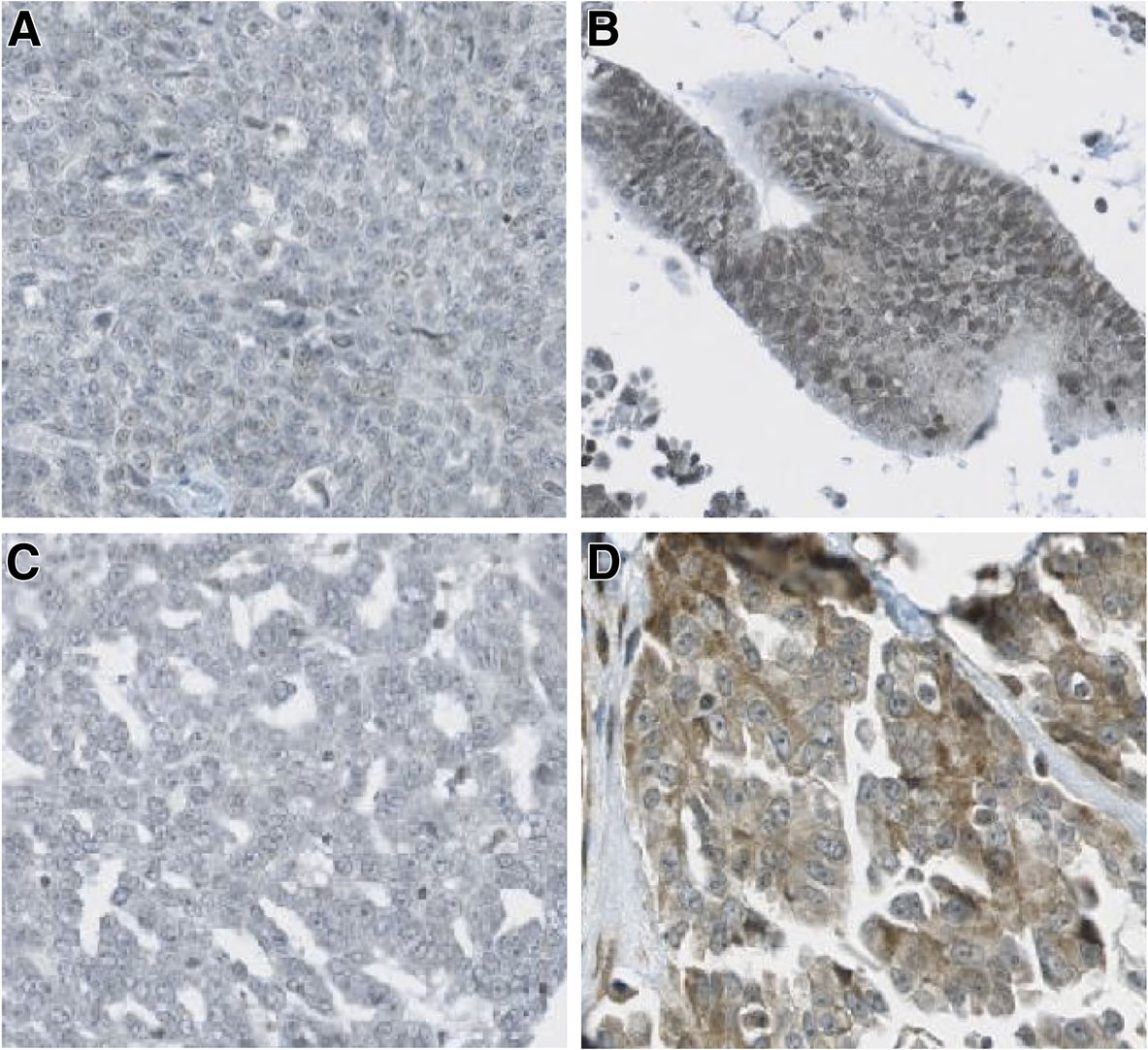
HtrA1 detection by immunohistochemistry. Negative nuclear staining (**a**) Positive nuclear staining (**b**) Negative cytoplasmic staining (**c**) Positive cytoplasmic staining (**d**)

**Fig. 4:**
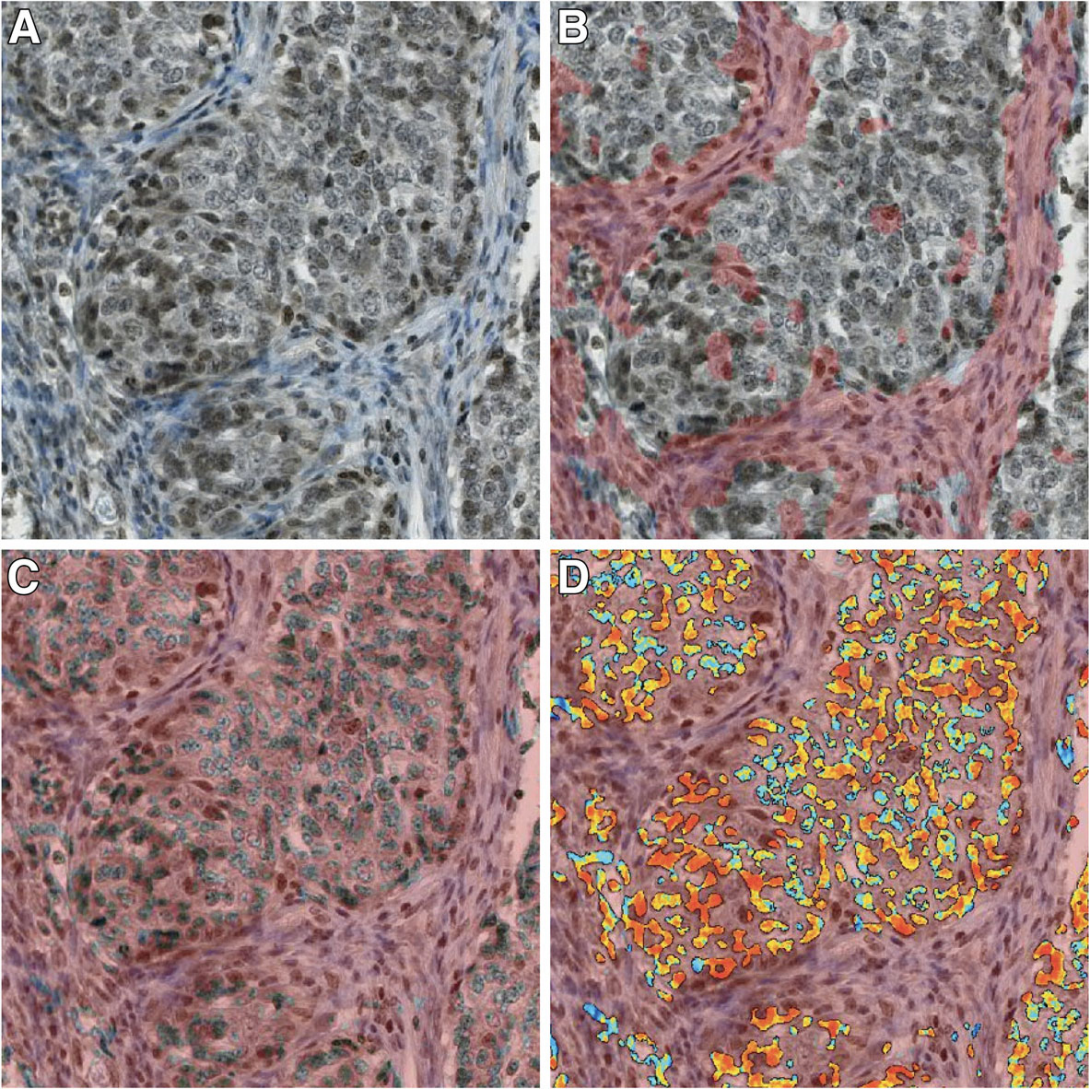
HtrA1 immunohistochemistry evaluated by digital analysis (example for nuclear staining). Initial digital image (**a**) Digital analysis removing stromal cells (areas removed from analysis in pink) (**b**) Digital analysis removing cytoplasm (**c**) Digital analysis of tumoral nuclei (color assessing the presence of staining) (**d**)

We demonstrated that the anti-HtrA1 antibody was specific (Fig. 2). Immunostaining of HtrA1 was observed both in the cytoplasm and in the nuclei of tumor cells (Fig. 3). By visual analysis, most tumor cells had positively stained cytoplasm (median: 100%, interquartile range: 60–100) while most nuclei were negative (median 10%, interquartile range: 2–28). Similar results were obtained by digital analysis in the cytoplasm (median: 73.2%, interquartile range: 56.2–86.4) and in the nuclei (median 36.4%, interquartile range: 16.2–59.7). A representative digital analysis mask is shown in Fig. 4. Accuracy between visual and digital evaluation was 61.5% for cytoplasmic HtrA1 expression and 75.4% for nuclear HtrA1 expression.

### HtrA1 expression in association with prognostic factors, progression or death

**Fig. 5:**
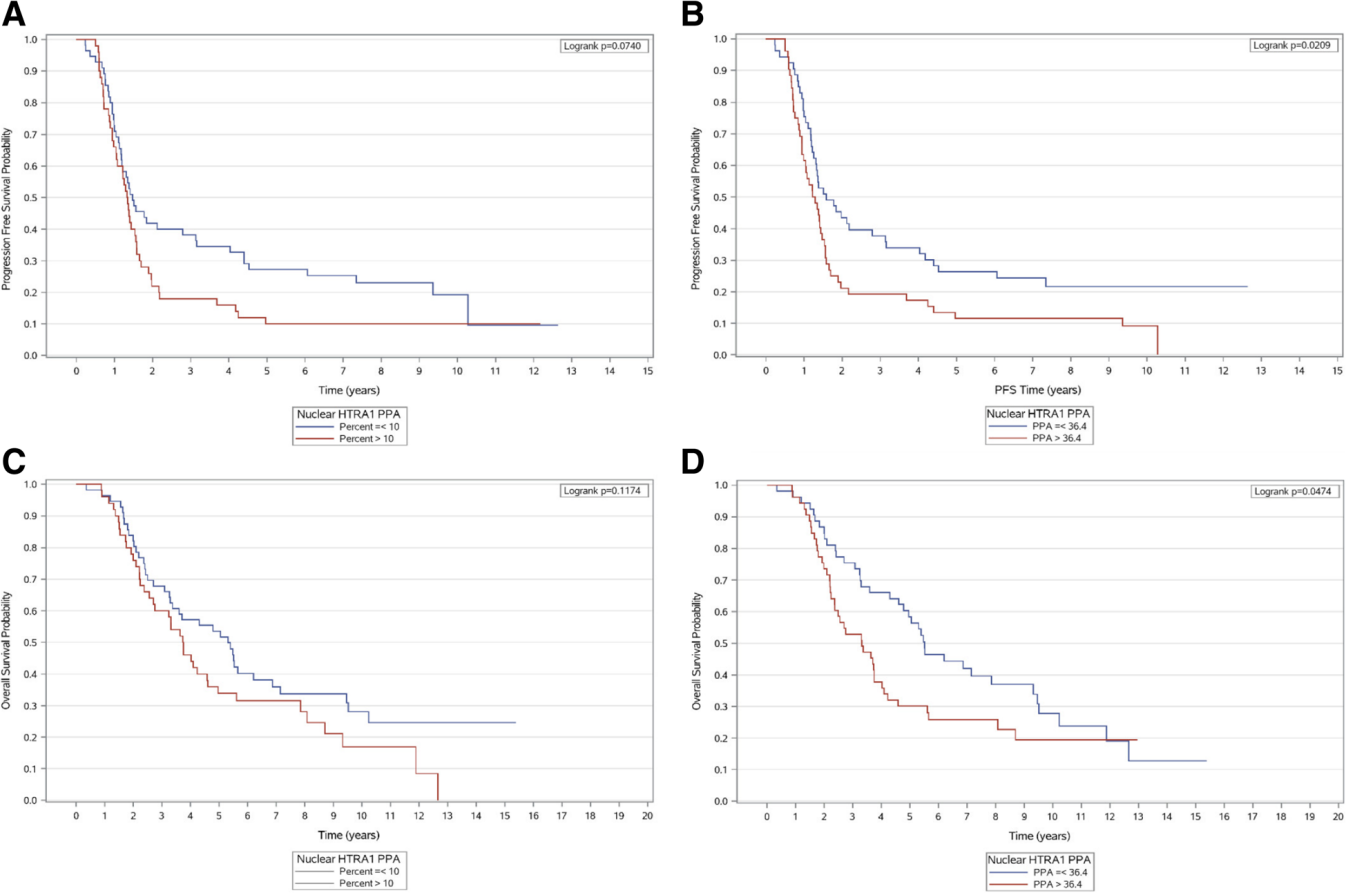
Kaplan-Meier curves showing association between progression and nuclear HtrA1 expression determined by visual (**a**) and digital (**b**) evaluation. Kaplan-Meier curves showing association between survival and nuclear HtrA1 expression determined by visual (**c**) and digital (**d**) evaluation

HtrA1 expression measured by digital evaluation (Table 2) or by visual evaluation (data not shown) was not associated with age, pre-operative serum CA-125 levels and FIGO stage. HtrA1 expression was significantly associated with prognosis only when HtrA1 was expressed in the nucleus (Table 3). Using Kaplan-Meier curves, we observed that low nuclear HtrA1expression was inversely associated with both progression (Fig. 5b) and death (Fig. 5d), when the assessment was done by digital evaluation. However the associations did not reach the statistical significance when the evaluation was visual (Fig. 5a and c). These associations were confirmed in multivariate analyses. Low HtrA1 nuclear expression was significantly associated with a lower risk of progression (digital evaluation, adjusted HR = 0.62 (0.40–0.95), *P* = 0.03). Low HtrA1 nuclear expression was also significantly associated with a lower risk of death (digital evaluation, adjusted HR = 0.60 (0.38–0.95), *P* = 0.03). The association with prognosis and death was of borderline statistical significance by visual assessment of HtrA1 (respectively, adjusted HR = 0.71 (0.46–1.09), *P* = 0.11 and 0.65 (0.41–1.04), *P* = 0.07) (Table 3).

**Table 2:** Associations between HtrA1 tumoral expression and standard prognostic factors

Characteristics	Nucleus			Cytoplasm		
	≤ 36.4%	> 36.4%	*P*-value	≤ 73.2%	> 73.2%	*P*-value
	*N* = 53 n (%)	*N* = 53 n (%)		*N* = 53 n (%)	*N* = 53 n (%)	
Age at diagnosis						
< 50	10 (18.9)	6 (11.3)	0.68	9 (17.0)	7 (13.2)	0.94
[50–60[	14 (26.4)	18 (34.0)		16 (30.2)	16 (30.2)	
[60–70[	16 (30.2)	16 (30.2)		16 (30.2)	16 (30.2)	
≥ 70	13 (24.5)	13 (24.5)		12 (22.6)	14 (26.4)	
FIGO stage						
I-II	5 (9.4)	3 (5.7)	0.46	4 (7.6)	4 (7.6)	1.00
III-IV	48 (90.6)	50 (94.3)		49 (92.4)	49 (92.4)	
CA125 (pmol/L)						
< 662	30 (56.6)	23 (43.4)	0.17	29 (54.7)	24 (45.3)	0.33
≥ 662	23 (43.4)	30 (56.6)		24 (45.3)	29 (54.7)

**Table 3:** Associations between HtrA1 tumoral expression and outcomes according to sub-cellular HtrA1 expression in women with high grade serous ovarian

Sub-cellular location	Methods, comparisons	Progression		Death	
		Crude HR (95% CI); *P*-value	Adjusted HR ^a^ (95% CI); *P*-value	Crude HR (95% CI); *P*-value	Adjusted HR ^a^ (95% CI); *P*-value
Nucleus	Visual ≤ 10.0 vs > 10.0	0.68 (0.45–1.04); 0.08	0.71 (0.46–1.09); 0.11	0.70 (0.45–1.10); 0.12	0.65 (0.41–1.04); 0.07
	Digital < 36.4 vs ≥ 36.4	0.61 (0.40–0.93); 0.02**	0.62 (0.40–0.95); 0.03**	0.64 (0.41–1.00); 0.05	0.60 (0.38–0.95); 0.03**
Cytoplasm	Visual < 100.0 vs 100.0	1.08 (0.71–1.64); 0.72	1.08 (0.71–1.64); 0.71	0.70 (0.45–1.10); 0.12	0.65 (0.41–1.04); 0.07
	Digital < 73.2 vs ≥ 73.2	0.84 (0.55–1.28); 0.41	0.82 (0.54–1.25); 0.35	0.90 (0.58–1.39); 0.62	0.84 (0.54–1.31); 0.43

*Abbreviations*: *HR* Hazard ratio, *CI* Confidence interval

^a^ Adjusted for FIGO stage (III-IV versus I-II), age at diagnosis (continuous) and pre-operative serum CA-125 levels (dichotomized according to the median value, ≥ 681.5 pmol/L versus < 681.5 pmol/L); ** P<0.05

## Discussion

Among the epithelial ovarian cancers, serous carcinoma is the most frequent histologic type [4]. Low grade and high grade serous carcinomas have been identified as subtypes with specific molecular characteristics [4]. In our study, we focused on a cohort of 106 women diagnosed with high-grade serous ovarian carcinoma. We observed that lower nuclear HtrA1 expression was associated with a lower risk of progression or death.

To our knowledge, this is the first study assessing the prognostic effect of HtrA1 in ovarian carcinoma. Narkiewicz et al. showed that there was no link between this protease and tumor grade, stage and histologic type, without testing the effects of HtrA1 on the prognosis of women with ovarian carcinoma [13]. Chien et al. focused on the response to cisplatin/cytoxan and cisplatin/taxol in 60 women with ovarian cancer. In these women, high expression of HtrA1, as detected by immunohistochemistry, was associated with a higher rate of complete and partial responses [14]. These results seem to be in opposition to our conclusions, since a better response might theoretically be associated with a better survival. However, major differences exist between our two cohorts, which prevent us from drawing any strong conclusion. First, these chemotherapy regimens have been replaced in our study by the new standard Taxol/Carboplatin regimen [20], which was received by 98% of women who underwent chemotherapy in our cohort. Second, in our cohort, the effects of HtrA1 expression on response could not be evaluated since only a few women (*n* = 17, 16%) did not experience a complete response to treatment according to the GCIG criteria [21, 22]. Therefore, we were unable to compare the effect of HtrA1 expressions with response to chemotherapy and to compare the effect of this response with progression or death. Furthermore, in above-mentioned studies, the association between HtrA1 and response was not assessed in a multivariate analysis and various histologic types of ovarian carcinomas were included. Further studies are needed to determine the relationship between HtrA1 expression, response to chemotherapy and its influence on prognosis.

Contrary to our results, the downregulation of HtrA1 in stomach, breast, and liver cancers has been associated with poor outcomes [14, 16–18]. Again, these data seem to contradict ours. However, in those studies, there was no mention as to whether the staining was nuclear or cytoplasmic. The function of HtrA1 could vary with regard to intracellular location. Three forms of HtrA1 have previously been identified, namely a precursor form (50 kDa) and two processing intermediates (38 and 29 kDa) and are expressed in either the cytoplasm or the nucleus [25]. The authors mention that the 29 kDa form, previously unidentified and which function is unknown, was predominant in the nucleus and that the 38 kDa from was present in the cytoplasm. The hypothesis that the forms of HtrA1 may have a different function was supported by Lorenzi et al. [26]. The authors reported, in urinary bladder cancer, that the 38 kDa form was significantly lower in cancer cells compared to normal tissue and that the same association was not observed with the 50 kDa form. The location of the protease could therefore explain our results, at least in part. Similarly, an association between the location of a biomarker and prognosis of ovarian cancer was also reported with Maspin, a protease inhibitor [27].

We strongly believe that our data are credible and strongly supported by our strict methodology. First, the images of HtrA1 immunostaining in the different publications are comparable to the immunostaining we obtained [14, 16–18]. Second, we validated the specificity of the anti-HtrA1 antibody that was used in our study, applying endogenous biotin blocking and verifying detection in 6 cell lines by Western blot analysis. We assessed the expression of HtrA1 in different cell compartments to improve our capacity to interpret results. Third, we performed a digital assessment which generated more precise data than visual inspection and provided stronger statistical power. We also imposed strict criteria for the assessment of clinical outcomes and the pathological evaluation of HtrA1 as each step was performed blindly to clinical information. Multivariate analyses were used to control for potential confounding factors.

HtrA1 is generally regarded as a tumor suppressor [28]. It has been suggested that HtrA1 plays a role in anoikis, a form of apoptosis that is induced when a cell loses normal cell-matrix interactions [9, 10, 29]. He et al. showed that HtrA1 forms a complex with X-linked inhibitor of apoptosis protein (XIAP) and degrades it via its protease activity [15]. XIAP degradation induces the production of caspase 3 and 7 which are used by caspase 9 in the intrinsic pathway for apoptosis [14, 15, 30]. The epidermal growth factor receptor (EGFR) pathway has also been reported to be activated in HtrA1-knockdown SKOV3 cells [9]. Other roles apart from the pro-apoptotic functions of HtrA1 are also emerging. HtrA1 has been described to activate the mammalian target of rapamycin (mTOR) pathway by degrading tuberin, a product of the tumor suppressor gene TSC2 [31]. How these in vitro properties can parallel our immunohistochemistry results still needs to be established.

## Conclusion

In conclusion, our results suggest that low nuclear expression of HtrA1 is associated with a lower risk of progression and death in women with high grade serous ovarian carcinoma. Further study is required to validate our findings with an independent cohort of women with serous ovarian carcinoma.

## Abbreviations

aHR: Adjusted hazard ratios
CHU de Québec: Centre Hospitalier Universitaire de Québec
CI: Confidence interval
FFPE: Formalin-fixed and paraffin-embedded
GCIG: Gynaecologic Cancer InterGroup
Htra1: High temperature requirement factor A1
RECIST: Response Evaluation Criteria in Solid Tumors
RT: Room temperature
TMA: Tissue microarrays
