# Correction to: HtrA1 expression and the prognosis of high-grade serous ovarian carcinoma: a cohort study using digital analysis

**DOI:** 10.1186/s13000-018-0748-2

**Published:** 2018-09-17

**Authors:** Andréanne Gagné, Bernard Têtu, Michèle Orain, Stéphane Turcotte, Marie Plante, Jean Grégoire, Marie-Claude Renaud, Isabelle Bairati, Dominique Trudel

**Affiliations:** 1 Laval University Cancer Research Center, Hôtel-Dieu-de-Québec, Centre Hospitalier Universitaire (CHU) de Québec, 11 Côte du Palais, Québec, QC G1R 2J6 Canada; 2 Anatomic Pathology and Cytology Department, Hôpital du St-Sacrement, Centre Hospitalier Universitaire (CHU) de Québec, Laval University, 1050 Chemin Ste-Foy, Québec, QC G1S 4L8 Canada; 3 Gynecologic Oncology Division, Centre Hospitalier Universitaire (CHU) de Québec, L’Hôtel-Dieu-de-Québec, 11 Côte du Palais, Québec, QC G1R 2J6 Canada; 4 Department of Pathology, Hôpital Saint-Luc, Centre Hospitalier Universitaire de Montréal, 058, rue Saint-Denis, Montréal, QC H2X 3J4 Canada; 5 The Research Centre of the University of Montreal Teaching Hospital (CR-CHUM)/Montreal Cancer Institute, 900 Rue St-Denis, Montreal, QC H2X 0A9 Canada; 6 Department of Pathology and Cellular Biology, University of Montreal, 2900, boulevard Édouard-Montpetit, Montreal, QC H3T 1J4 Canada; 7 Department of Pathology, Hôpital du St-Sacrement, Centre Hospitalier Universitaire de Québec, 1050, Chemin Ste-Foy, Québec, QC G1S 4L8 Canada

## Correction to: BMC Diagnostic Pathology (2018) 13:57 10.1186/s13000-018-0736-6

It has been highlighted that the original article [1] contained a typesetting mistake in the family name of Dominique Trudel. It was incorrectly captured as Trueel. This Correction article clarifies the correct family name of the author. The original article has been updated.

## References

[1] Gagné A (2018). HtrA1 expression and the prognosis of high-grade serous ovarian carcinoma: a cohort study using digital analysis. BMC Diagn Pathol.

